# Continuous photoproduction of hydrocarbon drop-in fuel by microbial cell factories

**DOI:** 10.1038/s41598-019-50261-6

**Published:** 2019-09-23

**Authors:** Solène Moulin, Bertrand Légeret, Stéphanie Blangy, Damien Sorigué, Adrien Burlacot, Pascaline Auroy, Yonghua Li-Beisson, Gilles Peltier, Fred Beisson

**Affiliations:** Aix Marseille University, CEA, CNRS, BIAM, EBM-Heliobiotec, Saint-Paul-lez-Durance, F-13108 France

**Keywords:** Enzymes, Metabolic engineering

## Abstract

Use of microbes to produce liquid transportation fuels is not yet economically viable. A key point to reduce production costs is the design a cell factory that combines the continuous production of drop-in fuel molecules with the ability to recover products from the cell culture at low cost. Medium-chain hydrocarbons seem ideal targets because they can be produced from abundant fatty acids and, due to their volatility, can be easily collected in gas phase. However, pathways used to produce hydrocarbons from fatty acids require two steps, low efficient enzymes and/or complex electron donors. Recently, a new hydrocarbon-forming route involving a single enzyme called fatty acid photodecarboxylase (FAP) was discovered in microalgae. Here, we show that in illuminated *E. coli* cultures coexpression of FAP and a medium-chain fatty acid thioesterase results in continuous release of volatile hydrocarbons. Maximum hydrocarbon productivity was reached under low/medium light while higher irradiance resulted in decreased amounts of FAP. It was also found that the production rate of hydrocarbons was constant for at least 5 days and that 30% of total hydrocarbons could be collected in the gas phase of the culture. This work thus demonstrates that the photochemistry of the FAP can be harnessed to design a simple cell factory that continuously produces hydrocarbons easy to recover and in pure form.

## Introduction

Increasing costs of petroleum extraction and environmental concerns over petroleum production and use have emphasized the need to develop renewable transportation fuels using energy-rich biomolecules such as alcohols, fatty acids and hydrocarbons^1,2^. Production of high titers of such carbon-based molecules has been achieved using heterotrophic or photosynthetic microbes but several impediments to commercialization of advanced biofuels remain^3–8^. For instance, some biomolecules like butanol can only be used as blending agents to conventional diesel fuel^9^ and others such as fatty alcohols or free fatty acids would require extraction from the fermentation broths. Still others, like fatty acid glycerol esters (oils), require expensive and energy-costly steps consisting in biomass harvest, conversion to biocrude and refinement, or oil extraction and chemical transesterification^10^.

In order to reduce drastically the costs of an industrial process, direct synthesis of fuel-like molecules by microbes combined with their release and capture outside of the cells is highly desired. One of the most promising types of fuel-like molecules appears to be hydrocarbons (HCs), especially *n*-alkanes, which are major components of current fossil fuels. Indeed, these compounds and their unsaturated analogs (*n*-alkenes) can be derived from abundant cell components (fatty acids) and, unlike oils, they are volatile or semi-volatile in the C5-C16 chain length range, which may be key to promote their release outside cells^11–13^. Formation of C9-C17 alka(e)nes by genetically modified microorganisms expressing HC-forming enzymes from a variety of organisms has been reported in bacterial and yeast cells^14–29^. Despite these advances, industrial prospects of microbial HC production seem limited by the competition of various host reductases for fatty intermediates of many HC-forming enzymes^14,17,21,30^ and the low turnover and complex cofactor requirement of the HC biosynthesis enzymes discovered so far^15,26,31–34^. Besides, recovery and quantification of liquid HCs in the gas phase of the cultures was only addressed in a single study but without estimation of productivity (mg L^−1^ h^−1^)^17^.

Recently, a novel HC-forming pathway has been discovered in the microalga *Chlorella variabilis* NC64A^35^. The HC-forming enzyme has been identified and shown to be a flavin-bearing protein that converts free fatty acids (FFA) into alkanes or alkenes^36^. This enzyme has been named fatty acid photodecarboxylase (FAP, EC 4.1.1.106) because it catalyzes the decarboxylation of free fatty acids (FFAs) using light (blue photons) to drive the reaction. It is thus a photoenzyme, a rare type of catalyst^37^. *In vitro*, the Chlorella FAP is known to accept a broad range of FFAs from C2 to C22^36,38^. FAP is likely to be of great interest for production of fuel in microorganisms because the one step conversion of fatty acids into HCs does not involve any intermediates such as aldehydes. In addition, FAP will not compete with other enzymes for chemical energy or cofactors as the reaction is electron-neutral and uses only light as energy source. A recent comparison of FAP with the widely used cyanobacterial aldehyde deformylating oxygenase (ADO) has shown that heterologous expression of the former in cyanobacteria increased HC production by 19-fold compared to the latter^39^. However, the potential of FAP to produce volatile HCs in the gas phase of cell cultures has not yet been investigated. Here our purpose is to address three questions: (i) What is the production rate of HCs in the gas phase of cell cultures expressing a HC-forming enzyme (compared to the production in cells)? (ii) Are HCs produced only transiently or is it possible to obtain a sustained production? (iii) What is the influence of light intensity on HC production in a FAP-expressing bacterial factory?

## Results

### Light dependency of FAP-based hydrocarbon production in *E. coli*

**Figure 1:**
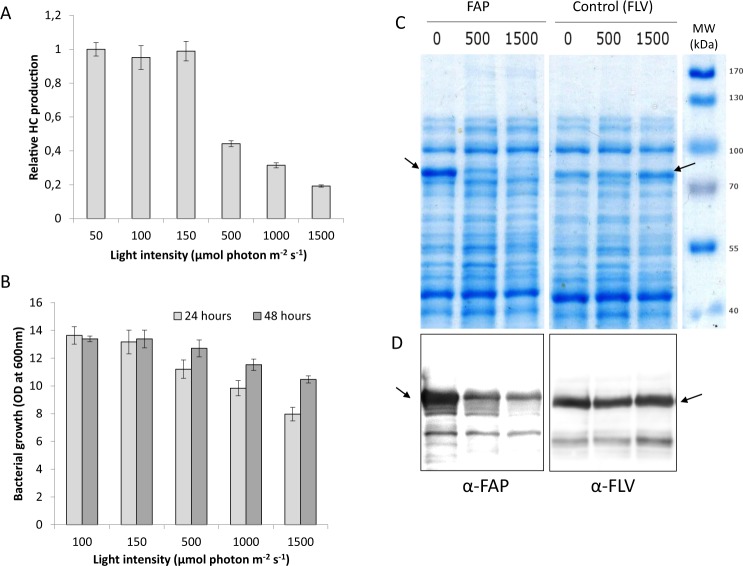
Influence of light intensity on HC production, bacterial growth and level of soluble FAP in a FAP-expressing *E. coli* strain. Strains were grown in batch culture under various light intensities. Data represent the mean ± SD of three independent experiments using three different bacterial cultures. (**A**) Relative HC production measured in cells 48 hours after induction. Production per culture volume was normalized by maximum obtained. (**B**) Growth of the culture measured by optical density (OD) at 600 nm, 24 hours or 48 hours post-induction. (**C**) SDS-PAGE of soluble proteins extracted from an *E. coli* strain expressing the FAP or a control strain expressing another flavoprotein (flavodiiron protein, FLV). 0, 500 and 1500 correspond to light intensities in µmol photons m^2^ s^−1^. Equal protein amounts were loaded in each lane. Bands of FAP and FLV are indicated by arrows. (**D**) Western blot of the SDS-PAGE revealed with anti-FAP or anti-FLV antibodies. Full-length gel and blots are included as Supplemental Fig. 2.

Because fatty acid photodecarboxylase (FAP) is a photoenzyme and its activity increases with white light intensity^36^, light may be a limiting factor of HC production in *E. coli*. HC production was analysed in batch cultures of an *E. coli* strain expressing FAP exposed to various white light intensities in order to determine saturating light. While this strain did not produce measurable HC amounts in the dark, growing cells under low to medium light intensity (50 to 150 µmol photons m^−2^ s^−1^) triggered maximal HC production **(**Fig. 1A**)**. Increasing light intensity up to 500 µmol photons m^−2^ s^−1^ resulted in a decreased production of HCs per liter of culture medium, which was even more drastic at 1000 and 1500 µmol photons m^−2^ s^−1^. Clearly, this drop in HC yield was correlated with a strong decrease in the growth of the bacterial culture **(**Fig. 1B**)**. Measurement of the dioxygen content in the culture medium using either a Clark electrode or a Membrane Inlet Mass Spectrometry (MIMS) showed that a quick decrease in dioxygen concentration occurred when the TB medium was exposed to blue light **(**Supplemental Fig. 1**)**. Depletion of dioxygen in the medium culture was thus likely to be responsible for reduction of *E. coli* growth under high light. Interestingly, HC production at 48 hours post-induction dropped by 50% although cell growth dropped only by 20%. This indicated that cell growth was not the only cause of the drop in HC production under high light. We therefore checked the level of FAP expression by immunodetection in the soluble proteins extracted from *E. coli* cells exposed to different light intensities. FAP amounts present in the *E. coli* FAP-expressing strain decreased with increasing light intensity whereas the flavodiiron protein (FLV), another flavoprotein used as a control, remained constant **(**Fig. 1C,D and Supplemental Fig. 2**)**. This result shows that under high light the reduction in growth of *E. coli* cultures does not affect protein expression under the T7 promotor and suggests that he FAP is likely photo-damaged when exposed to an excess of light. Since the FAP catalytic cycle involves a radical form of FAD^36^, a lack of substrate and an excess of light may indeed lead to accumulation of free radicals and inactivation of the FAP. Taken together, these experiments therefore show that in an *E. coli* FAP-based HC production system, maximum productivity is reached at relatively low light irradiance.

### Set-up of a system to grow bacteria expressing FAP under light and collect hydrocarbons

**Figure 2:**
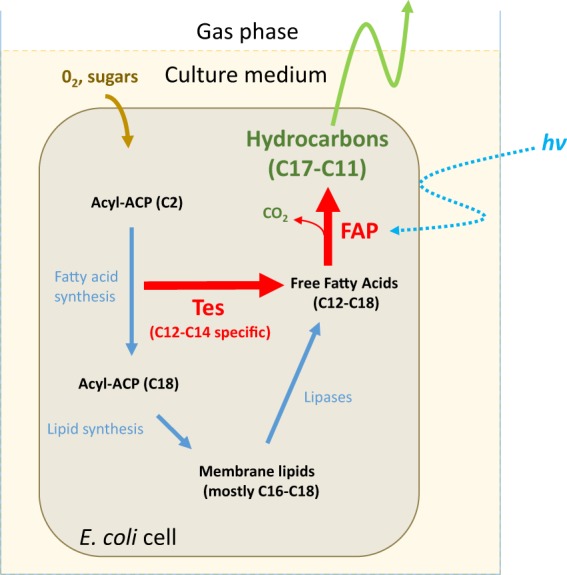
Metabolic reactions used for photoproduction of hydrocarbons by *E. coli* cells. Two strains were cultivated. One was transformed with a gene coding a fatty acid photodecarboxylase (FAP) from *Chlorella*. The other one was transformed with genes coding FAP and a medium-chain specific thioesterase (Tes) from *Umbellularia*. Both genes are expressed under inducible promotors. FAP requires light as a cofactor. ACP: acyl carrier protein.

**Figure 3:**
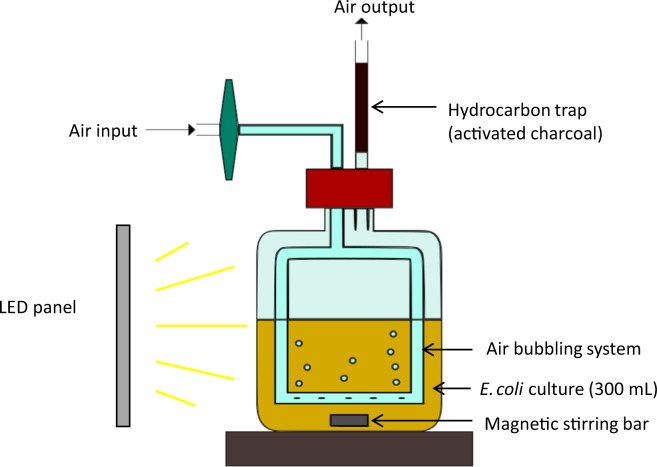
Scheme of the cultivation system used for photoproduction of hydrocarbons by *E. coli* cells. Air input was set at 15 L h^−1^ because this flow rate allowed proper oxygenation of the culture while minimizing foaming (too much foam resulted in partial wetting of the hydrocarbon trap). In controls experiments, a second trap connected on the outlet of the first one allowed to show that potential losses represented less than 1% of total hydrocarbons found in the first trap. Light intensity was about 100 µmol photon m^2^ s^−1^ in the culture, corresponding to 500 µmol photon m^2^ s^−1^ incident light.

With a view to design the simplest cell factory producing a drop-in fuel that could be easily recovered, we assessed the potential of FAP to produce medium-chain *n*-alkanes and *n*-alkenes (C9-C13) in the gas phase of the cultures. A strain coexpressing the FAP and a fatty acid thioesterase from the plant *Umbellularia californica* (Tes) was constructed **(**Fig. 2**)**. The thioesterase Tes interrupts fatty acid elongation by hydrolyzing specifically medium-chain fatty acids esterified to acyl carrier protein and releasing medium-chain free fatty acids^40^. In order to investigate production of HCs by the FAP on a larger scale, *E. coli* cells were cultivated in transparent glass bottles bubbled with air and illuminated with medium-light intensity white LED panels **(**Fig. 3**)**. In larger scale cultures, each cell is subjected to a fluctuating light due to constant agitation and the shading effect. Therefore, incident light cannot be easily correlated with actual light received by cells. We thus set up the incident light, so that the light intensity measured in the cell culture was within the optimal range determined on the smaller scale cultures (50–150 μmol photons m^−2^ s^−1^). An incident light intensity of 500 µmol photons m^−2^ s^−1^ was chosen because it was found to result in an illumination of around 100 µmol photons m^−2^ s^−1^ in the bulk of the cell culture. To quantify the fraction of alka(e)nes present in the gas phase of the culture, a HC-trapping device consisting of a low-cost matrix used in industry (activated charcoal) was placed at the gas outlet of the cultivation device. This system thus allowed to investigate HC production in cells as well as the gas phase of *E. coli* strains expressing the FAP.

### Combination of FAP and Tes allows continuous production of volatile hydrocarbons

**Figure 4:**
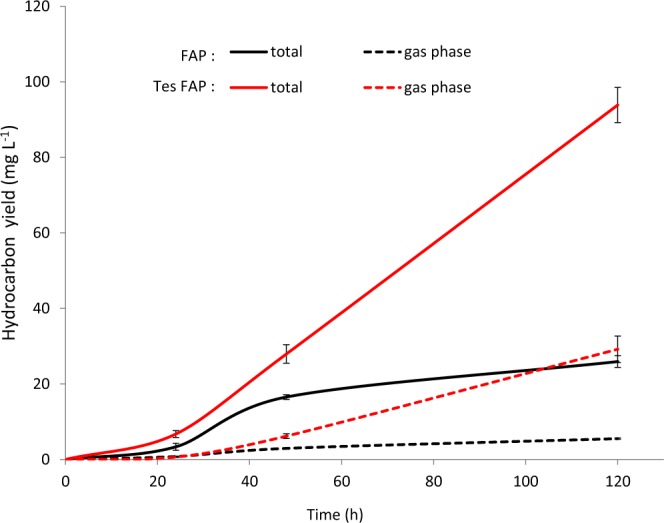
Hydrocarbon production in cultures of *E. coli* expressing FAP or FAP and thioesterase. Time course of hydrocarbon production. Cells were grown in batch culture under constant light illumination. *Chlorella variabilis* FAP (FAP) and *Umbellularia californica* thioesterase (Tes) were expressed in *E. coli*. Hydrocarbons were quantified in the harvested cells and in the gas phase of the culture using the hydrocarbon trap (Fig. 2). Total refers to cells plus gas phase. Data represent the mean ± SD of three independent experiments using three different bacterial cultures.

**Figure 5:**
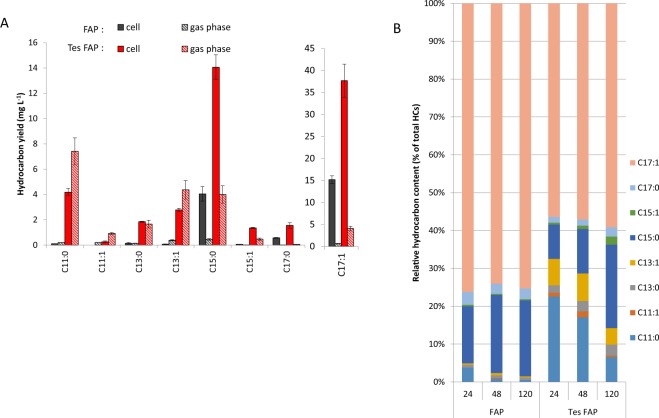
Profile of hydrocarbons produced in *E. coli* cultures. Production of *E. coli* strains expressing FAP (FAP) or co-expressing FAP and thioesterase (Tes FAP) (**A**) Profile of hydrocarbons produced in *E. coli* cultures 120 h after induction. (**B**) Relative hydrocarbons composition of cell content in function of time (hours). Data represent the mean ± SD of three independent experiments using three different bacterial cultures.

The FAP of *C. variabilis* was first expressed alone under a lactose-inducible promotor. Following induction of FAP expression, HC production reached a pseudo-plateau within two days, with 22 mg L^−1^ of HCs in the cell fraction and 26 mg L^−1^ in total **(**Fig. 4**)**. HCs produced were mainly composed of C15:0 alkane and C17:1 alkene (20% and 75% respectively of the total HC production), with shorter chain HCs present in smaller amounts. Not surprisingly, the gas phase was enriched in shorter HCs, 45% of the total being C11 and C13 HCs **(**Fig. 5**)**. As the culture aged, the relative content of short-chain HCs in the cells decreased, which was consistent with the idea that shorter HCs diffused to the gas phase.

**Figure 6:**
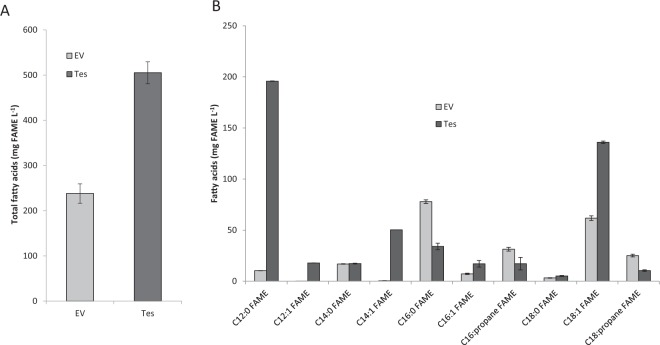
Fatty acid content in *E. coli* strain expressing *Umbellularia californica* thioesterase. (**A**) Total fatty acid content. (**B**) Content in individual fatty acids. After whole cell transmethylation, cellular fatty acids were quantified as fatty acid methyl esters (FAME) and expressed as mg FAME per liter of culture. A strain expressing a pBAD empty vector (EV) and a strain expressing pBAD-Thioesterase (Tes) were analyzed. Data represent the mean ± SD of three independent experiments using three different bacterial cultures.

In order to boost the production of medium-chain HCs, the FAP was then coexpressed with the thioesterase Tes. The gene encoding Tes was driven by an arabinose-inducible promoter to avoid overproduction of free fatty acids. When expressed alone in *E. coli*, Tes induced a 2-fold increase in the total fatty acid content of cells **(**Fig. 6A**)**, which was mainly due to an increase in C12:0 and C14:1 but also C18:1 fatty acids **(**Fig. 6B**)**. This showed that Tes was effective at enriching cells in medium-chain fatty acids and boosting fatty acid synthesis in general. Coexpression of Tes with FAP resulted in a striking 4-fold increase in HC production compared to the strain expressing only FAP, reaching a titer of 94 mg L^−1^ at day 5 **(**Fig. 4**)**. Production of HCs in gas phase also significantly increased to 29 mg L^−1^ of HC, representing 30% of total HC production. HC profile showed an increase in medium-chain HC species with 15% of the production in the cell comprised of C11-C13 HCs compared to less than 2% without Tes **(**Fig. 5B**)**. In the gas phase, the percentage of C11:0 alkane reached more than 30% of total HCs and C11 to C13 HCs represented more than 60% of the total HC production. Most importantly, the production rate from day one to day two reached 0.88 mg L^−1^ h^−1^ and remained constant at 0.91 mg L^−1^ h^−1^ from day two to day five. In comparison, without the thioesterase, production reached 0.55 mg L^−1^ h^−1^ until day two but it dropped down to 0.13 mg L^−1^ h^−1^ between day two and five. Therefore, HC production in a strain expressing only the FAP stopped as the bacteria reached stationary phase. By contrast, in the presence of the heterologous expressed thioesterase, fatty acid synthesis was kept active even in stationary phase, presumably to keep constant the pool of acyl-ACPs, which was continuously depleted by conversion to FFAs and HCs.

## Discussion

Here we describe a simple FAP-based cell factory that continuously releases volatile hydrocarbons and we quantify the production rates in the cells and in the gas phase of the cultures. We also show that low to medium light intensities (50–150 µmol photons m^−2^ s^−1^) allow to reach maximal HC productivity and that higher irradiances result in decreased FAP amounts.

Despite little metabolic engineering, HC productivity measured for the FAP-Tes strain (0.91 mg L^1^ h^−1^) was in the same range as the best ones that could be estimated for alkanes/alkenes production from previous studies, i.e. around 1–2 mg L^−1^ h^−1 ^^14,20^. Additional genetic modifications such as arrest of fatty acid β-oxidation or increase of FFA pool^17,41^ may further boost HC production. But the key point is made here that the thioesterase from *Umbellularia californica* not only enriches FAP products in medium-chain HCs that are volatile, but is also key to achieve a continuous production of HCs while cells are not dividing anymore. Besides, the absence of aldehyde intermediates allows to recover HCs in pure form and not as a blend with fatty alcohols and fatty aldehydes^14,17^.

This work therefore shows that coexpression in *E. coli* of the newly discovered photoenzyme FAP and a medium-chain thioesterase results in a light-driven synthesis of medium-chain alkanes and alkenes, which can be easily recovered from the gas phase of bacterial culture in substantial amounts and highly pure form. Most importantly, evidence is provided that the production of volatile HCs by bacterial cells can be maintained at a high level over a period of several days. Therefore, this study demonstrates that the combination of FAP and thioesterase is a simple but powerful tool to turn *E. coli* into a cell factory that continuously releases volatile HCs. It should thus provide useful information for a bio-based production of fuel-range HCs on a larger scale.

## Methods

### Strains and cultivation conditions

*E. coli* BL21 pRIL strain was used throughout this study. Strains obtained by transformation of BL21 cells are listed in Supplemental Table 1. Pre-cultures were incubated at 37 °C overnight in Luria-Bertani (LB) broth medium with the following antibiotics, kanamycin 50 µg mL^−1^, chloramphenicol 34 µg mL^−1^, ampicillin 100 µg mL^−1^, depending on strain used. Cultures were performed in 24-deepwell plates for light intensity analysis, in 100 mL erlens for FAP expression analysis and in 500 mL bottles with a bubbling system for volatile HC analysis (containing 4, 30 or 300 mL of culture medium respectively). Light was provided by white LED panels. Terrific Broth (TB) medium (12 g L^−1^ tryptome, 24 g L^−1^ yeast extract, 12.5 g ^−1^ K_2_HPO_4_, 2.3 g L^−1^ KH_2_PO_4_) with addition of required amounts of antibiotics was inoculated using a pre-culture (dilution 1:50) and grown at 37 °C at 180 rpm. When optical density (OD) at 600 nm reached around 0.8, cultures were induced by addition of isopropyl β-D-1-thiogalactopyranoside (IPTG) at a final concentration 0.5 mM or/and by addition of arabinose at a final concentration of 0.2% (w/v). Upon induction, temperature was lowered to 22 °C and culture were illuminated with white LED panels set at various light intensities (50 to 1500 µmol photon m^2^ s^−1^ incident light). For cultures in bottles, a stirring bar and an air bubbling system allowed mixing as well as gas exchange of the culture, air flow was run continuously at 15 L h^−1^ and the air outlet was connected to cartridges containing activated charcoal to trap HCs on exit.

### Gene synthesis and construction of plasmids

Synthetic genes codon-optimized for *E. coli* were designed for *Chlorella* fatty acid photodecarboxylase (FAP) (Sorigué *et al*. 2017) and for the thioesterase (Tes) from the plant *Umbellularia californica* (Voelker and Davis 1994). Sequences of FAP and Tes corresponded to the proteins without their predicted chloroplast transit peptide **(**Supplemental Fig. 3**)**. The plasmid expressing FAP was constructed from pLIC07 by Golden Gate cloning using *Bsa*I restriction enzyme **(**Supplemental Fig. 4**)**. pLIC07 is a vector derived from pET28 by insertion of *SacB* gene with restriction site for ligation independent cloning (LIC) **(**Supplemental Fig. 3**)**. For co-expression, the plasmid expressing Tes was constructed from pBAD/Myc-his B by In-Fusion®. Primers used for plasmid construction are listed in Supplemental Table 2.

### Extraction of hydrocarbons and fatty acids

For quantification of HCs contained inside the cells, one mL of culture was pelleted by centrifugation in glass tubes. Transmethylation was conducted by adding 2 mL of methanol containing 5% (v/v) sulfuric acid to the cell pellet. Internal standards (10 µg of hexadecane and 20 µg of triheptadecanoylglycerol) were added for quantification. Reaction was carried out for 90 min at 85 °C in sealed glass tubes. After cooling down, one mL of 0.9% (w/v) NaCl and 500 µL of hexane were added to the samples to allow phase separation and recovery of fatty acid methyl esters (FAMEs) and HCs in the hexane phase. Samples were mixed and then centrifuged to allow phase separation. One µL of the hexane phase was injected in the GC-MS/FID. For quantification of HCs trapped in the air outlet (i.e. the gaseous phase of the culture), the activated charcoal was incubated at room temperature in 2 mL of hexane containing10 µg of hexadecane as an internal standard. Samples were centrifuged for 10 min to pellet charcoal. One µL of the hexane phase was injected in the GC-MS/FID.

### GC-MS/FID analyses

Analyses were carried out on an Agilent 7890A gas chromatographer coupled to an Agilent 5975 C mass spectrometer (simple quadrupole). A Zebron 7HG-G007-11 (Phenomenex) polar capillary column (length 30 m, internal diameter 0.25 mm, and film thickness 0.25 mm) was used. Dihydrogen carrier gas was at 1 mL min^−1^. For samples obtained from whole cell transmethylations, oven temperature was programmed with an initial 2-min hold time at 35 °C, a first ramp from 35 to 170 °C at 15 °C min^−1^, followed by 1-min hold time at 170 °C then a second ramp from 170 to 240 °C at 5 °C min^−1^ and a final 2-min hold time at 240 °C. For samples recovered from activated charcoal, oven temperature was programmed with an initial 2-min hold time at 35 °C, a first ramp from 35 to 180 °C at 15 °C min^−1^ followed by 2-min hold time at 180 °C. Samples were injected in splitless mode (1 min) at 250 °C. The MS was run in full scan over 40 to 350 amu (electron impact ionization at 70 eV), and peaks of FAMEs and HCs were quantified based on the FID signal using the internal standards C17:0 fatty acid and hexadecane respectively.

### Protein analysis

Total proteins were extracted from cell pellets using a Bugbuster® protein extraction reagent. After 30 min extraction at room temperature, the supernatant was denaturated by incubation at 70 °C for 20 min in NuPAGE LDS sample buffer with 1 mM dithiothreitol. Total protein extract were separated on a 15% (w/v) acrylamide Bis-Tris gel using a Tris Glycine SDS buffer. After staining with ProSieve^TM^ EX Safe Stain, quantification of total protein content in gel was done using an infrared imaging scaner measuring 700 nm fluorescence. For immunoblot analysis, proteins were loaded on a constant protein basis, separated as described before and then transferred with a semi-dry technic onto a BioTrace NT nitrocellulose membrane (Sigma-Aldrich). Membrane was blocked overnight at 4 °C by incubation with 5% (w/v) dried milk in Tris Buffer Saline containing 0.1% (w/v) Tween 20. Membrane was then incubated at room temperature for 2 h with specific primary polyclonal antibodies from rabbit (dilution 1:2000) and then for 1 h with a secondary anti-rabbit antibody coupled to horseradish peroxidase (HRP). Immobilon ^TM^ Western Chemiluminescent HRP substrate (EMD Millipore) was used for detection and images were recorded using a G:BOX Chemi XL (Syngene).

### Dioxygen measurement

O_2_ exchanges were measured using a Clark-type electrode (S1 dioxygen electrode, Hansatech), or a Membrane inlet mass spectrometry system^42^. Blue (460 nm) or red (620 nm) light was applied on top of the cuvette using a Dual-PAM 100 module (Walz). The Clark electrode was set so that ambient O_2_ concentration in water (236 µmol l^−1^) gives a signal of 1 V, and absence of O_2_ (reached using reduced dithionite) gives a signal of 0 V. O_2_ concentration was recorded continuously upon red or blue illumination in both systems for a few minutes.

## Supplementary information

supplemental information
