# Supplementary material for: Continuous photoproduction of hydrocarbon drop-in fuel by microbial cell factories
Source: Sci Rep. 2019 Sep 23;9:13713. doi: 10.1038/s41598-019-50261-6 (PMC6757031; doi:10.1038/s41598-019-50261-6)
Supplement: Supplementary file 1 — supplemental information [file 41598_2019_50261_MOESM1_ESM.pdf]

Supplemental information for Moulin et al.

Continuous photoproduction of hydrocarbon drop-in fuel by microbial cell factories

Solène Moulin[1], Bertrand Légeret[1], Stéphanie Blangy[1], Damien Sorigué[1], Adrien Burlacot[1,] Pascaline Auroy[1], Yonghua Li-Beisson[1], Gilles Peltier[1], Fred Beisson[1*]

[1]Aix Marseille University, CEA, CNRS, BIAM, EBM-Heliobiotec, Saint-Paul-lez-Durance, F-13108 France.

Supplemental Table 1 : List of strains used for metabolic engineering

Strain	Relevant genotype
BL21	E. coli B F⁻ ompT hsdS(r_B^- m_B^-) dcm⁺ Tet^r gal λ(DE3) endA Hte [*argU ileY leuW* Cam^r]
FAP	pLIC07 : FAP in BL21
EV	pBAD/Myc-his B (empty vector) in BL21
Tes	pBAD/Myc-his B : Tes in BL21

Supplemental Table 2: List of primers used.

Primer	Sequence	Gene
pLIC07-FAP-F	TTGGTCTCCCAATG GCCAGCGCAGTTGAAGATATTC	short version FAP (*Chlorella variabilis*)
pLIC07-FAP-R	TTGGTCTCCTTAC TCA TGCTGCAACGGTTGCCGG	
pBAD-Tes-F	GAGGAATTAACCATGTTAGAATGGAAACCAAAACCAA	Thioesterase (*Umbellularia californica*)
pBAD-Tes-R	GAGTTTTTGTTCTAGAACACGTGGTTCAGCTGGAATAACTGA	

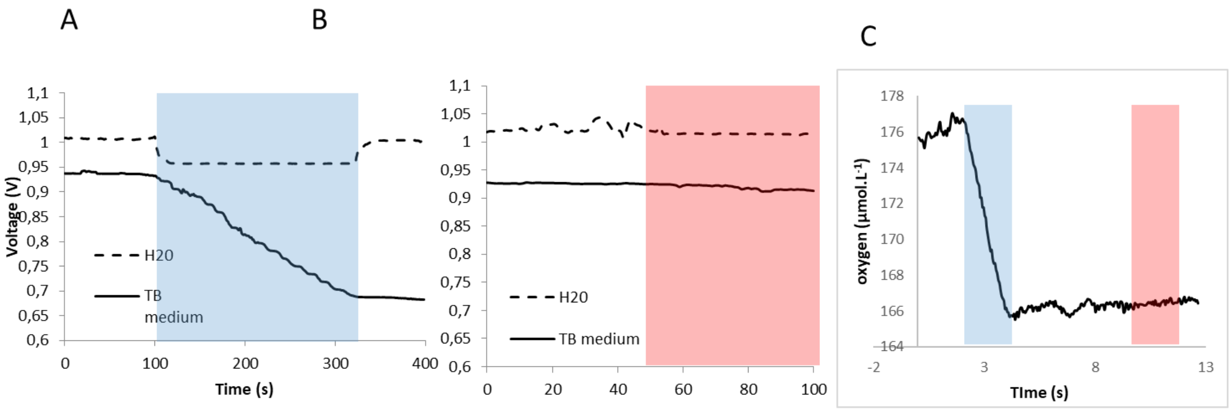

Supplemental Figure 1 : Light induced O_2 consumption by TB medium. Concentration of O_2 in TB medium was recorded upon illumination by red and blue light. (A,B) O_2 in TB and water upon blue (A) and red (B) light exposure (700 µmol$_{photon}$.m^{-2}.s^{-1}) measured by a Clark-type electrode. (C) O_2 measurement in TB upon blue and red light exposure (600 µmol$_{photon}$.m^{-2}.s^{-1}) measured by a MIMS.

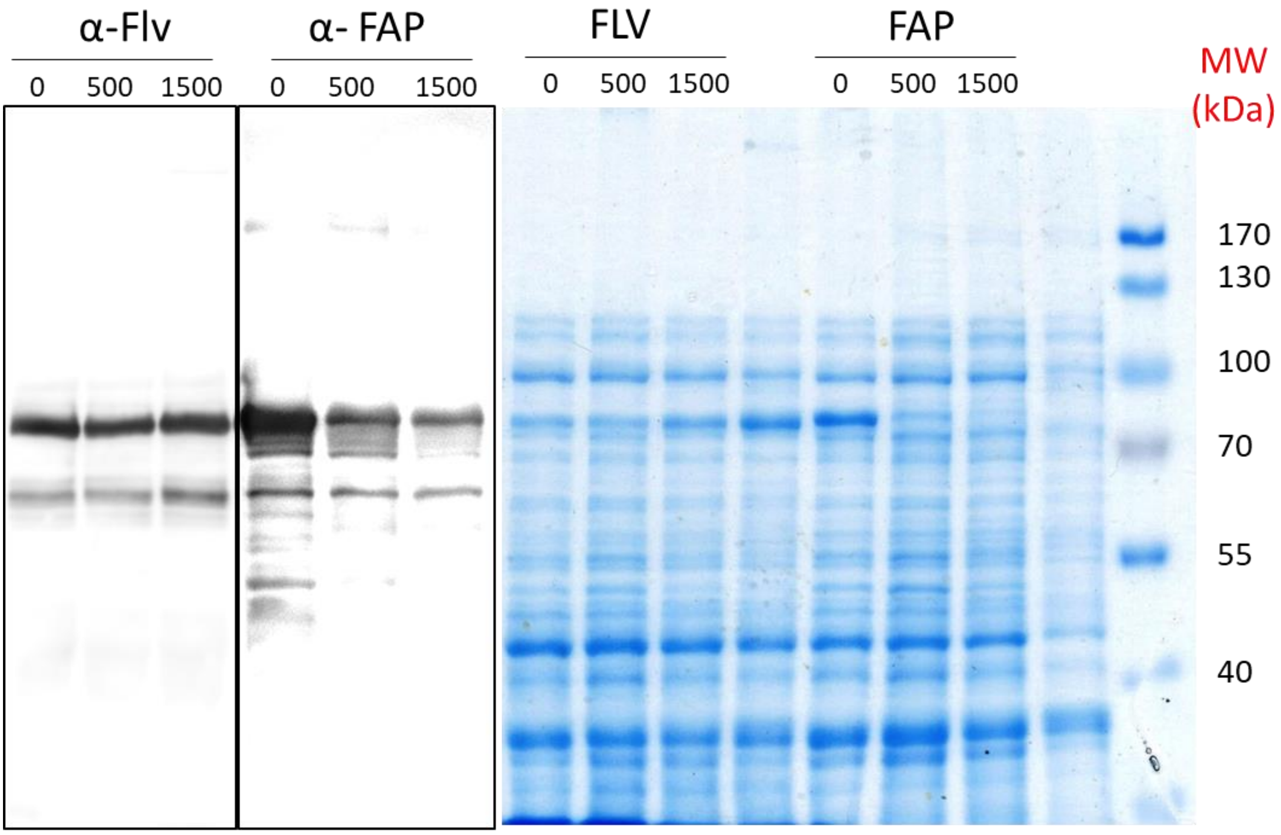

Supplemental Figure 2: Uncropped versions of blots and gels of Fig.1

(A)

GCCAGCGCAGTTGAAGATATTCGTAAAGTTCTGAGCGATAGCAGCAGTCCGGTTGCAGGTCAGAA
ATATGATTATATTCTGGTTGGTGGTGGCACCGCAGCATGTGTTCTGGCAAATCGTCTGAGCGCAGA
TGGTAGCAAACGTGTTCTGGTTCTGGAAGCAGGTCCGGATAATACCAGCCGTGATGTTAAAATTCC
GGCAGCAATTACCCGTCTGTTTCGTAGTCCGCTGGATTGGAACCTGTTTAGCGAACTGCAAGAACA
GCTGGCAGAACGTCAGATTTATATGGCACGTGGTCGTCTGCTGGGTGGTAGCAGCGCAACCAATGC
AACCCTGTATCATCGTGGTGCAGCCGGTGATTATGATGCATGGGGTGTTGAAGGTTGGAGCAGCGA
AGATGTTCTGAGCTGGTTTGTTCAGGCAGAAACCAATGCAGATTTTGGTCCGGGTGCATATCATGG
TAGCGGTGGTCCGATGCGTGTTGAAAATCCGCGTTATACCAATAAACAGCTGCATACCGCATTTTT
CAAAGCAGCAGAAGAAGTTGGTCTGACCCCGAATAGCGATTTTAATGATTGGAGCCATGATCATGC
AGGTTATGGCACCTTTCAGGTTATGCAGGATAAAGGCACCCGTGCAGATATGTATCGTCAGTATCT
GAAACCGGTTCTGGGTCGTCGTAATCTGCAGGTTCTGACCGGTGCAGCAGTTACCAAAGTTAATAT
TGATCAGGCAGCAGGTAAAGCACAGGCACTGGGTGTTGAATTTTCAACCGATGGTCCGACCGGTGA
ACGTCTGAGTGCAGAACTGGCACCGGGTGGTGAAGTTATTATGTGTGCCGGTGCAGTTCATACCCC
GTTTCTGCTGAAACATAGCGGTGTTGGTCCGAGCGCAGAACTGAAAGAATTTGGTATTCCGGTTGT
TAGCAATCTGGCAGGCGTTGGTCAGAATCTGCAGGATCAGCCTGCATGTCTGACCGCAGCACCGGT
TAAAGAAAAATATGATGGTATTGCCATCAGCGATCACATTTATAACGAAAAAGGTCAGATTCGCAA
ACGTGCAATTGCAAGCTATCTGCTGGGAGGTCGTGGTGGTCTGACCAGTACCGGTTGTGATCGTGG
TGCATTTGTTCGTACCGCAGGTCAGGCACTGCCGGATCTGCAGGTACGTTTTGTTCCGGGTATGGCA
CTGGATCCGGATGGTGTTAGCACCTATGTTCGTTTTGCAAAATTTCAGAGCCAGGGTCTGAAATGG
CCGAGCGGTATTACCATGCAGCTGATTGCATGTCGTCCGCAGAGCACCGGTAGCGTTGGTCTGAAA
AGCGCAGATCCGTTTGCACCGCCTAAACTGAGTCCGGGTTATCTGACCGATAAAGATGGTGCAGAT
CTGGCAACCCTGCGTAAAGGTATTCATTGGGCACGTGATGTTGCACGTAGCAGCGCACTGAGCGAA
TATCTGGATGGTGAACTGTTTCCGGGTAGCGGTGTTGTTAGTGATGATCAGATTGATGAATATATCC
GTCGCAGCATTCATAGCAGCAATGCAATTACCGGCACCTGTAAAATGGGTAATGCCGGTGATAGCA
GCAGCGTTGTTGATAATCAGCTGCGTGTTCATGGTGTGGAAGGTCTGCGTGTTGTTGATGCAAGCG
TTGTTCCGAAAATTCCGGGTGGTCAGACAGGTGCACCGGTTGTTATGATTGCAGAACGTGCAGCAG
CACTGCTGACCGGTAAAGCAACCATTGGTGCAAGCGCAGCAGCACCGGCAACCGTTGCAGCATGA

(B)

ATGTTAGAATGGAAACCAAAACCAAAATTACCACAATTATTAGATGATCACTTTGGTTTACACGGT
TTAGTTTTCCGTCGTACATTTGCTATTCGTTCATATGAAGTTGGTCCAGATCGTTCAACATCAATTTT
AGCTGTTATGAATCACATGCAAGAAGCTACATTAAACCACGCTAAATCAGTTGGTATTTTAGGTGA
TGGTTTTGGTACAACATTAGAAATGTCAAAACGTGATTTAATGTGGGTTGTTCGTCGTACACATGTT
GCTGTTGAACGTTATCCAACATGGGGTGATACAGTTGAAGTTGAATGTTGGATTGGTGCTTCAGGT
AATAATGGTATGCGTCGTGATTTCTTAGTTCGTGATTGTAAAACAGGTGAAATCTTAACACGTTGTA
CATCATTATCAGTTTTAATGAATACACGTACACGTCGTTTATCAACAATTCCAGATGAAGTTCGTGG
TGAAATTGGTCCAGCTTTTATTGATAATGTTGCTGTTAAAGATGATGAAATTAAAAAATTACAAAA
ATTAAACGATTCAACAGCTGATTATATTCAAGGTGGTTTAACACCACGTTGGAATGATTTAGATGTT
AATCAACACGTTAATAACTTAAAATACGTTGCTTGGGTTTTTGAAACAGTTCCAGATTCAATTTTTG
AATCACACCACATTTCATCATTTACATTAGAATATCGTCGTGAATGTACACGTGATTCAGTTTTACG
TTCATTAACAACAGTTTCAGGTGGTTCATCTGAAGCTGGTTTAGTTTGTGATCACTTATTACAATTA
GAAGGTGGTTCTGAAGTTTTACGTGCTCGTACAGAATGGCGTCCAAAATTAACTGATTCTTTTCGTG
GTATTTCAGTTATTCCAGCTGAACCACGTGTT

Supplemental Figure 3: Nucleotide sequences of synthetic genes used for FAP (A) and Tes (B)

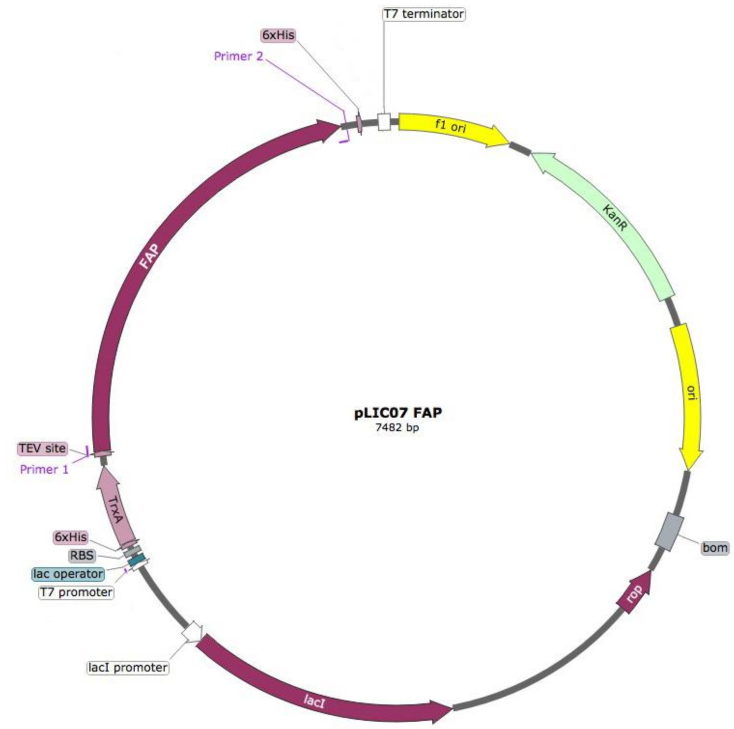

Supplemental Figure 4: Plasmid map for FAP expression in pLIC07 vector
